# Characterising Maturation of GFP and mCherry of Genomically Integrated Fusions in *Saccharomyces cerevisiae*

**DOI:** 10.21769/BioProtoc.2710

**Published:** 2018-01-20

**Authors:** Sviatlana Shashkova, Adam JM Wollman, Stefan Hohmann, Mark C Leake

**Affiliations:** 1 Biological Physical Science Institute, Departments of Physics and Biology, University of York, York, UK; 2 Department of Chemistry and Molecular Biology, University of Gothenburg, Göteborg, Sweden; 3 Department of Biology and Biological Engineering, Chalmers University of Technology, Göteborg, Sweden

**Keywords:** Single-molecule, Fluorescence, Fluorescent protein maturation, Protein fusion, GFP, mCherry, Yeast

## Abstract

Single-molecule fluorescence microscopy enables unrivaled sub-cellular quantitation of genomically encoded fusions of native proteins with fluorescent protein reporters. Fluorescent proteins must undergo *in vivo* maturation after expression before they become photoactive. Maturation effects must be quantified during single-molecule analysis. Here we present a method to characterise maturation of GFP and mCherry genetic protein fusions in budding yeast *Saccharomyces cerevisiae*.

## Background

Single-molecule fluorescence microscopy enables sensitive quantification of molecular stoichiometry, mobility and copy number, not only on a cell-by-cell basis but also precisely to individual sub-cellular compartments (Leake, 2012; Wollman and Leake, 2015; Shashkova *et al*., 2017). The technique relies on endogenously expressed fluorescent protein fusions of the wild type protein of interest such that there is one-to-one labelling. However, all fluorescent proteins have an *in vivo* maturation time varying from a few minutes to several tens of minutes before entering a bright fluorescing state (Badrinarayanan *et al.*, 2012). It is therefore of upmost importance to measure any maturation effects and quantify if there is any immature ‘dark fraction’ of labelled protein. These measurements are also particularly relevant to fluorescence recovery after photobleaching (FRAP). FRAP can be used to study molecular turnover in living cells (Beattie *et al*., 2017). FRAP is based on photobleaching of a cell region where a fluorescently labelled component is localized, followed by quantification of any fluorescence recovery in that region over time. The measured relation between the fluorescence intensity as a function of time following an initial photobleach can be used to determine molecular mobility and kinetics parameters, such as the rate of dissociation of a particular fluorescent component from a molecular complex (Leake *et al*., 2006). Therefore, any ‘new’ fluorescence coming from fluorescent protein maturation might affect this apparent result. We present here a protocol to characterise the maturation of Mig1-GFP and Nrd1-mCherry fusion proteins in living yeast *Saccharomyces cerevisiae* cells used in our single-molecule studies (Wollman *et al*., 2017).

We blocked protein translation in living cells by adding cycloheximide (Hartwell *et al*., 1970), and then measured any cellular fluorescence recovery after cells were completely photobleached by continuous illumination. Such fluorescence recovery is then used as a metric for newly matured GFP and/or mCherry in the cell. Our results are broadly consistent with *in vivo* maturation of GFP and mCherry reported previously (Badrinarayanan *et al*., 2012; Khmelinskii *et al*., 2012), but since maturation kinetics may be dependent on cell type and the specific extracellular microenvironment, it is important to quantify these maturation effects under the same experimental conditions used for the *in vivo* microscopy on the actual fusion strains of interest.

## Materials and Reagents

1. Sterile pipette tips, 1 ml, 200 µl, 10 µl (STARLAB, catalog numbers: S1111-6801, S1111-0806, S1111-3800)
2. 14 ml conical tubes (Corning, Falcon^®^, catalog number: 352059)
3. Petri dishes 92 mm diameter (Thermo Fisher Scientific, Thermo Scientific™, catalog number: 172931)
4. Microscopy slides (Fisher Scientific, catalog number: FB58622)
5. Cover slips (Scientific Laboratory Supplies, catalog number: MIC3124)
6. Yeast *S. cerevisiae* YSH2348 with Mig1-GFP and Nrd1-mCherry genetically integrated protein fusions, *MATa MIG1-GFP-HIS3 NRD1-mCherry-hphNT1 MET LYS* (Hohmann lab, University of Gothenburg, Sweden)
7. D(+)-Glucose (VWR, catalog number: 101176K)
8. Bacto-yeast extract (BD, Bacto™, catalog number: 212750)
9. Peptone from meat (Merck, catalog number: 1072241000)
10. Agar-agar (Merck, catalog number: 1016141000)
11. MilliQ water
12. Yeast nitrogen base without amino acids, without (NH_4_)_2_SO_4_ (Sigma-Aldrich, catalog number: Y1251)
13. Ammonium sulfate, (NH_4_)_2_SO_4_ (Merck, catalog number: 1012171000)
14. Complete supplement mixture (ForMedium, catalog number: DCS0019)
15. Cycloheximide (Sigma-Aldrich, catalog number: C7698)
16. EtOH (VWR, catalog number: VWRC20821.330)
17. Glucose 50% w/v (see Recipes)
18. YPD agar with 4% glucose (see Recipes)
19. YPD liquid medium with 4% glucose (see Recipes)
20. YNB (Yeast Nitrogen Base) liquid medium without glucose (see Recipes)
21. YNB (Yeast Nitrogen Base) liquid medium with 4% glucose (see Recipes)
22. 100 mg/ml cycloheximide solution in EtOH (see Recipes)

## Equipment

1. Pipettes (STARLAB, model: ErgoOne^®^ Single-Channel Pipette, 2-20 µl, 20-200 µl and 100-1,000 µl)
2. pH-meter (Scientific & Chemical Supplies, catalog number: PHM975050)
3. Two timers (Fisher Scientific, catalog number: 15177414)
4. Autoclave (Getinge, model: 400/500LS-E Series Steam Sterilizers (533LS-E))
5. Magnetic stirrer (Chemtech Scientific, model: C-MAG HS7)
6. 30 °C incubator (Eppendorf, New Brunswick Scientific™, model: Innova^®^ 4000)
7. Spectrophotometer (Biochrom, model: WPA S800)
8. Centrifuge (Eppendorf, model: 5810 R)
9. Mercury-arc excitation fluorescence microscope Zeiss Axiovert 200M (Carl Zeiss, model: Axiovert 200 M) with an AxioCamMR3 camera with separate filter sets: 38HE for GFP and 43HE for mCherry excitation; Plan-Apochromat 1.40-numerical-aperture oil immersion, 100x objective

## Software

1. AxioVision
2. ImageJ 1.50g
3. Excel
4. MATLAB 2017a

## Procedure

**Figure 1:**
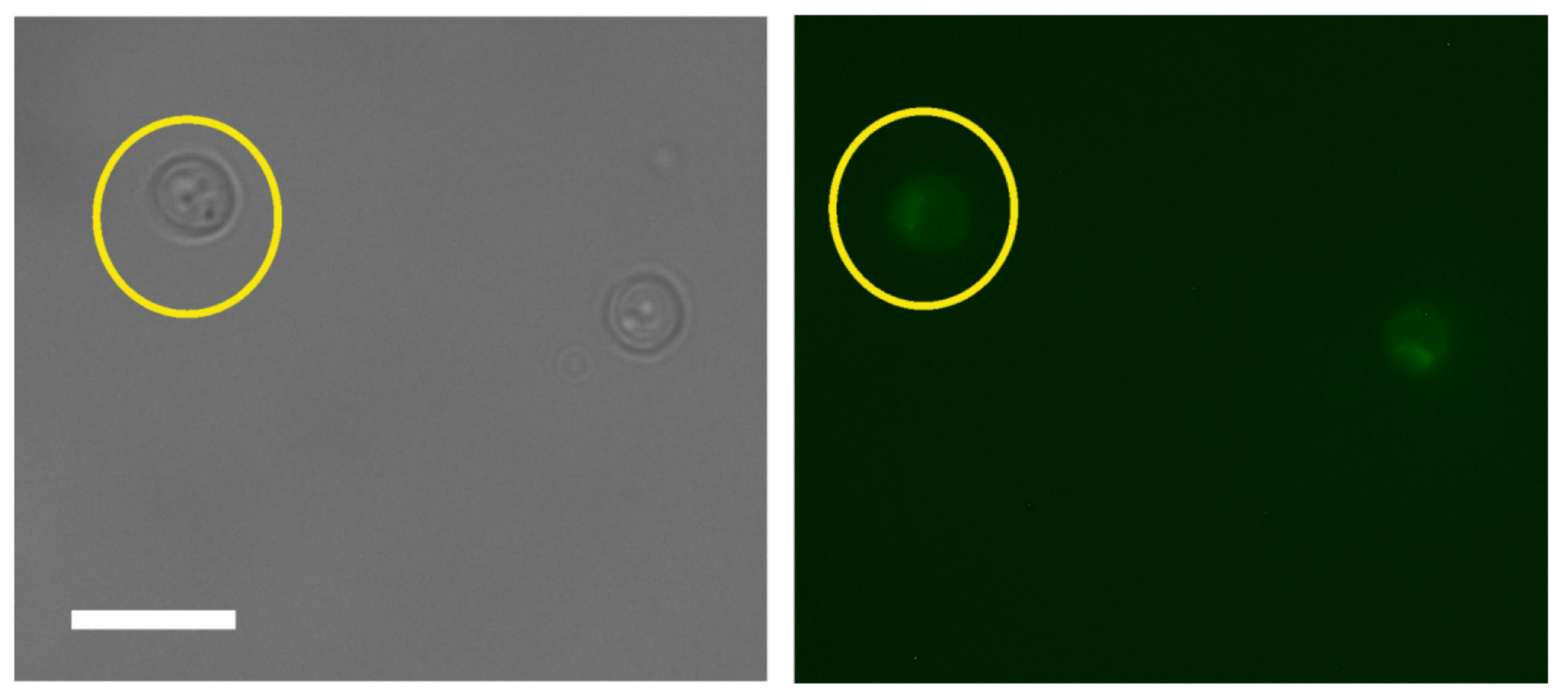
Selection of the region of interest for cell measurements. Scale bar = 20 µm.

**Figure 2:**
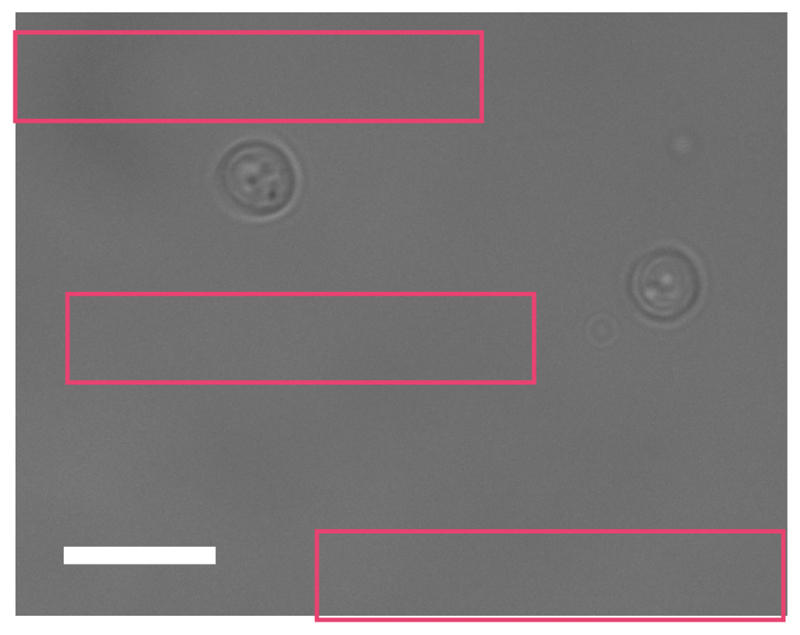
Selection of the region of interest for the background measurements. Scale bar = 20 µm.

**Figure 3:**
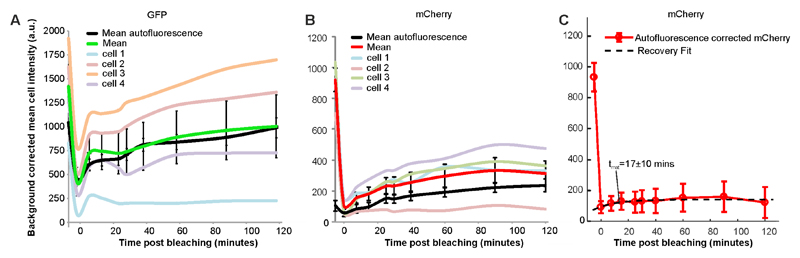
Characterisation of GFP and mCherry maturation times *in vivo*. GFP maturation within genomically integrated protein fusion (A). Maturation of a genomically integrated mCherry fusion (B) and its’ exponential recovery fit (C). Time normalized to bleach time at t = 0.

- Cell preparation
  1. Streak cells from a frozen stock, using a sterile pipette tip on a freshly-prepared YPD agar plate (see Recipes), and incubate at 30 °C for at least 24 h.
  2. Set an overnight culture in a 14 ml tube by inoculating 3 ml of YPD with cells grown on a YPD plate. Single colonies are not needed for genomically integrated strains. Incubate at 30 °C, 180 rpm.
  3. In the morning exchange the YPD medium (see Recipes) to YNB medium (see Recipes) supplemented with 4% glucose:
    - Pellet the cells by centrifugation at 1,000 *x g* for 3 min, remove the supernatant.
    - Resuspend the cells in 3 ml of YNB medium without any carbon source.
    - Pellet the cells by centrifugation at 1,000 *x g* for 3 min, remove the supernatant.
    - Suspend the cells in 3 ml of YNB supplemented with 4% glucose and incubate at 30 °C, 180 rpm, for ~4 h.
    - Wash the culture by centrifugation (1,000 *x g*, 3 min) and re-suspend in 2 ml of YNB with 4% glucose. Incubate at 30 °C, 180 rpm, for about 10 min.
    - Add 2 μl of 100 mg/ml cycloheximide solution (see Recipes) to the final concentration of 100 μg/ml. Incubate for 1 h at room temperature, without shaking, protect from light.
    - Place 5 μl of the culture on a microscope slide and cover with a 22 x 22 mm coverslip. Avoid any air under the coverslip.
- Data acquisition
  1. Place the sample under the microscope, coverslip on the objective and find a region of interest containing 5-10 cells (100x magnification) which appear stationary and firmly anchored to the glass surface.
  2. Optimize exposure times for *in vivo* imaging of both GFP and mCherry fluorescent proteins to be able to detect a clear signal without saturating the detector. Under our microscope: GFP exposure time–22 sec, mCherry–7 sec.
  3. Find another region with 5-10 cells positioned far away from the previous one to avoid any potential bleaching from previous illumination exposure.
  4. Take a brightfield and a fluorescence image, by pressing the ‘snap’ button, with both channels using chosen exposure times, opening the mercury lamp shutter for only the length of exposure.
  5. Photobleach GFP or mCherry by continuous illumination of the appropriate wavelength until the region appears completely dark. Continue for 1 min longer. With our settings the total exposure time is: 3 min 40 sec for GFP and 4 min for mCherry. Immediately after, begin timing and acquire a picture of the bleached fluorescent protein with an appropriate channel and a brightfield image. This is denoted time point 0 min.
  6. Continue acquiring both fluorescent and brightfield pictures at the following time points after bleaching: 7.5, 15, 25, 30, 40, 60, 90 and 120 min.
  7. As simultaneous photobleaching of GFP and mCherry is not possible under this microscope, the time points were staggered for GFP and mCherry as listed in Table 1.
- Data analysis
  1. Images are converted into open standard tiff files from zvi by AxioVision software.
  2. Further analysis is performed using ImageJ.
    - Open the first unbleached brightfield image.
    - By choosing an ‘oval’ selection tool, define a region of interest (ROI), an area around a cell as shown in Figure 1. It does not matter how much of non-cell area is included as every cell will be background-correct during the analysis.
    - Open a fluorescence image of the same set, and define the same area of the same cell by simultaneously choosing ‘Shift’ and ‘E’ keys on the keyboard (Selection → Restore).
    - From the menu bar select: Analyze → set measurements. Pick ‘area’ (represents a number of pixels, *N*) and ‘integrated density’ (sum intensity for the cell, *S_cell_*). Press ‘OK’.
    - To obtain numeric values press ‘Ctrl’ + ‘M’ (Analyse → Measure). Record the result in Excel.
    - Repeat throughout the entire data set for both channels keeping the same ROI.
    - Repeat the entire procedure for all cells.
    - Background correction: Choose random background areas around cells (Figure 2) and obtain numerical results for sum intensity (*S_bg_*).
    - Find the average (*S_Abg_*) and multiply by the number of pixels from cell (*N*) measurements. This is the intensity of the background represented within the cell area (*I_bg_*).
      $$\begin{array}{l} S_{Abg}=\langle S_{bg}\rangle ;\\ I_{bg}=S_{Abg}\times N; \end{array}$$
    - Subtraction of the average background sum intensity (*I_bg_*) from the total intensity of the cell (*S_cell_*) represents *I_cell_*, the cellular fluorescence intensity with background correction.
      $$I_{cell}=S_{cell}-I_{bg}.$$
    - The average of fluorescence intensity of all cells analysed within the data set gives the final value of the fluorescence intensity (*I_final_*) with appropriate estimation of SD and/or SE.
      $$I_{final}=\langle I_{cell}\rangle .$$
    - Plot the final fluorescence intensity (*I_final_*) vs. experimental time (Figures 3A and 3B) for fluorescently labelled cells and wild type autofluorescent cells. Any signal above autofluorescence is due to fluorescent protein maturation. For GFP (Figure 3A), no maturation was detected so it can be assumed that all of the fluorescent protein was mature in the cells and there is no ‘dark’ fraction. For mCherry (Figure 3B), some fluorescence recovery was measured. The following steps outline quantification of the maturation time and dark fraction.
    - Subtract the autofluorescence from the mean fluorescent protein intensity at each time point (Figure 3C).
    - Export the intensity and time values after the bleach by copying and pasting into two new variables in MATLAB, called x (for the time values ) and y (intensity values):
      - Right click on the Workspace → New. Name it x or y. Press ‘Enter’ on the keyboard.
      - Double click on this new variable opens a table in the Editor where values of time (for x) or intensity (for y) can be pasted.
    - Open the curve fitting toolbox from the Apps menu.
    - Select x for ‘X data’ and y for ‘Y data’.
    - Choose custom equation and type:
      $$I_{rec}*\left(1-\exp\left(-\frac{x}{t_{mat}}\right)\right)+I_{bleach}$$
      where, *I_bleach_* is the remaining intensity after the bleach, *I_rec_* is the recovered intensity above *I_bleach_* and *t_mat_* is the maturation time.
    - If ‘Auto fit’ is ticked, fitting will be automatic.
    - If the fit has not converged correctly, adjust the ‘Start point’ parameters in ‘Fit Options’ to reasonable estimates from the data *i.e.*, y at x = 0 for *I_bleach_* and y at x = end minus *I_bleach_* for *I_rec_*.
    - If the fit has converged record the fit and goodness of fit parameters from the ‘Results’ panel. For Figure 3C, *I_rec_* = 4.3 x 104 ± 3 x 104 counts, *I_bleach_* = 5.2 x 104 ± 2.5 x 104 counts, *t_mat_* =17 ± 10 min with *R^2^* = 0.7.
    - To calculate the proportion dark, immature protein; divide *I_rec_* by the initial, autofluorescence corrected pre-bleach intensity. Here give ~5%.

## Data analysis

Data was analyzed as outlined in section C of the Procedure. Statistical methods are outlined in Wollman *et al.* (2017) but also briefly outlined here. In imaging experiments, each cell can be defined as a biological replicate sampled from the cell population. Sample sizes of ~10 cells were used to generate reasonable estimates of fluorescent protein maturation and are similar to previous studies (Badrinarayanan *et al*., 2012). Technical replicates are not possible with irreversible photobleaching however noise is characterized by the autofluorescent of wild type control cell measurements.

## Notes

Autofluorescence is calculated as indicated in the protocol above but using a wild type yeast strain (*i.e*., without any fluorescent proteins present).

## Recipes

1. Glucose 50% w/v
  Weigh 500 g of glucose
  Bring up to 1 L with MilliQ water
  Dissolve by using magnetic stirrer with heating
  Autoclave for 20 min at 121 °C
2. YPD agar with 4% glucose
  Mix yeast extract 5 g, Bacto-peptone (peptone from meat) 10 g and agar 10 g
  Bring up to 460 ml with MilliQ water
  Autoclave for 20 min at 121 °C
  Add 40 ml of glucose 50% w/v
  Cast plates: approximately 25 ml of the medium per plate
  Let them solidify, store upside down at 4 °C
3. YPD liquid medium with 4% glucose
  Mix yeast extract 5 g and Bacto-peptone (peptone from meat) 10 g
  Bring up to 460 ml with MilliQ water
  Autoclave for 20 min at 121 °C
  Add 40 ml of glucose 50% w/v
4. YNB (Yeast Nitrogen Base) liquid medium without glucose
  Mix yeast nitrogen base without amino acids, without (NH_4_)_2_SO_4_ 1.7 g, complete supplement
  0.79 g, (NH_4_)_2_SO_4_ 5 g
  Dissolve in 900 ml of MilliQ water, adjust pH 5.8-6.0 using NaOH
  Bring up to 1,000 ml with MilliQ water
  Autoclave for 20 min at 121 °C
5. YNB (Yeast Nitrogen Base) liquid medium with 4% glucose
  Mix yeast nitrogen base without amino acids, without (NH_4_)_2_SO_4_ 1.7 g, complete supplement
  0.79 g, (NH_4_)_2_SO_4_ 5 g
  Dissolve in 900 ml of MilliQ water, set pH 5.8-6.0 using NaOH
  Bring up to 920 ml with MilliQ water
  Autoclave for 20 min at 121 °C
  Add 80 ml of glucose 50% w/v
6. 100 mg/ml cycloheximide solution in EtOH
  Weigh 0.5 g of cycloheximide and dissolve in 5 ml of absolute EtOH
  Aliquot and store at -20 °C

## Figures and Tables

**Table 1:** Order of photobleaching and data acquisition using two channels

	GFP	mCherry
1	Bleaching for 3 min 40 sec	
2	Timer start, Time point 0 min	
3	Time point 7.5 min	
4		Bleaching for 4 min
5		Timer start, Time point 0 min
6	Time point 15 min	
7		Time point 7.5 min
8	Time point 25 min	
9		Time point 15 min
10	Time point 30 min	
11		Time point 25 min
12		Time point 30 min
13	Time point 40 min	
14		Time point 40 min
15	Time point 60 min	
16		Time point 60 min
17	Time point 90 min	
18		Time point 90 min
19	Time point 120 min	
20		Time point 120 min
